# Targeting and regulation of autophagy in hepatocellular carcinoma: revisiting the molecular interactions and mechanisms for new therapy approaches

**DOI:** 10.1186/s12964-023-01053-z

**Published:** 2023-02-09

**Authors:** Mehrdad Hashemi, Niloufar Nadafzadeh, Mohammad Hassan Imani, Romina Rajabi, Setayesh Ziaolhagh, Seyedeh Delaram Bayanzadeh, Raheleh Norouzi, Reihaneh Rafiei, Zeinab Khazaei Koohpar, Behnaz Raei, Mohammad Arad Zandieh, Shokooh Salimimoghadam, Maliheh Entezari, Afshin Taheriazam, Athanasios Alexiou, Marios Papadakis, Shing Cheng Tan

**Affiliations:** 1 Department of Genetics, Faculty of Advanced Science and Technology, Tehran Medical Sciences, Islamic Azad University, Tehran, Iran; 2 Farhikhtegan Medical Convergence Sciences Research Center, Farhikhtegan Hospital Tehran Medical Sciences, Islamic Azad University, Tehran, Iran; 3 Faculty of Veterinary Medicine, Science and Research Branch, Islamic Azad University, Tehran, Iran; 4 Department of Clinical Science, Faculty of Veterinary Medicine, Shahr-E Kord Branch, Islamic Azad University, Tehran, Chaharmahal and Bakhtiari Iran; 5 Department of Cell and Molecular Biology, Faculty of Biological Sciences, Tonekabon Branch, Islamic Azad University, Tonekabon, Iran; 6 Department of Food Hygiene and Quality Control, Division of Epidemiology, Faculty of Veterinary Medicine, University of Tehran, Tehran, Iran; 7 Department of Biochemistry and Molecular Biology, Faculty of Veterinary Medicine, Shahid Chamran University of Ahvaz, Ahvaz, Iran; 8 Department of Orthopedics, Faculty of Medicine, Tehran Medical Sciences, Islamic Azad University, Tehran, Iran; 9 Department of Science and Engineering, Novel Global Community Educational Foundation, Hebersham, Australia; 10 AFNP Med Austria, Vienna, Austria; 11 Department of Surgery II, University Hospital Witten-Herdecke, University of Witten-Herdecke, Heusnerstrasse 40, 42283 Wuppertal, Germany; 12 UKM Medical Molecular Biology Institute, Universiti Kebangsaan Malaysia, Kuala Lumpur, Malaysia

**Keywords:** Hepatocellular carcinoma, Autophagy, Chemoresistance, Metastasis, Stemness

## Abstract

Autophagy is an evolutionarily conserved process that plays a role in regulating homeostasis under physiological conditions. However, dysregulation of autophagy is observed in the development of human diseases, especially cancer. Autophagy has reciprocal functions in cancer and may be responsible for either survival or death. Hepatocellular carcinoma (HCC) is one of the most lethal and common malignancies of the liver, and smoking, infection, and alcohol consumption can lead to its development. Genetic mutations and alterations in molecular processes can exacerbate the progression of HCC. The function of autophagy in HCC is controversial and may be both tumor suppressive and tumor promoting. Activation of autophagy may affect apoptosis in HCC and is a regulator of proliferation and glucose metabolism. Induction of autophagy may promote tumor metastasis via induction of EMT. In addition, autophagy is a regulator of stem cell formation in HCC, and pro-survival autophagy leads to cancer cell resistance to chemotherapy and radiotherapy. Targeting autophagy impairs growth and metastasis in HCC and improves tumor cell response to therapy. Of note, a large number of signaling pathways such as STAT3, Wnt, miRNAs, lncRNAs, and circRNAs regulate autophagy in HCC. Moreover, regulation of autophagy (induction or inhibition) by antitumor agents could be suggested for effective treatment of HCC. In this paper, we comprehensively review the role and mechanisms of autophagy in HCC and discuss the potential benefit of targeting this process in the treatment of the cancer.

**Graphical abstract:**

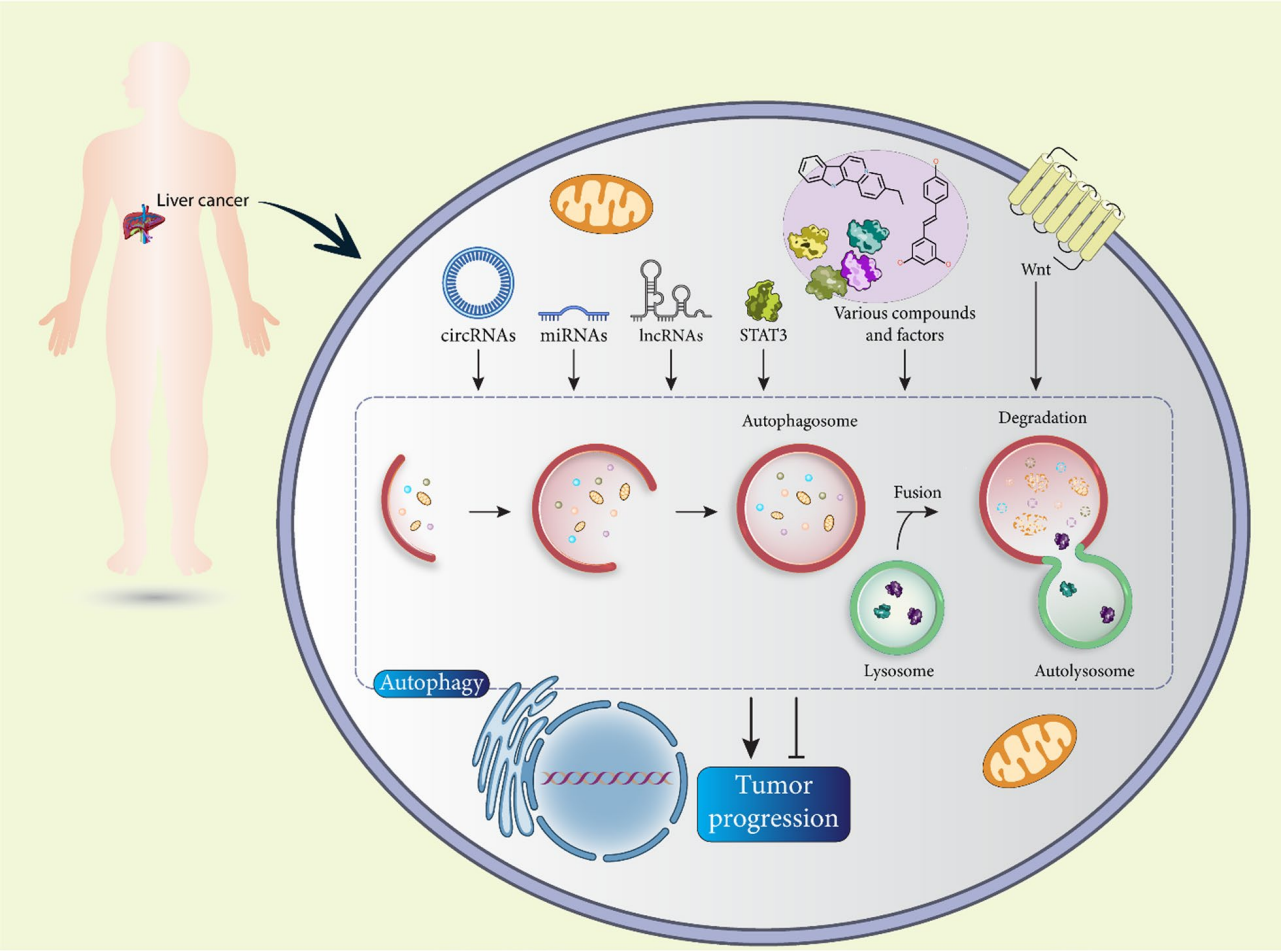

**Video Abstract**

**Supplementary Information:**

The online version contains supplementary material available at 10.1186/s12964-023-01053-z.

## Introduction

Autophagy is an evolutionarily conserved mechanism with a potential role in organelle and protein turnover that may also be involved in regulating metabolism and controlling cell quality [1, 2]. The process of autophagy is dependent on lysosomes and the main goal of autophagy is to provide nutrients and energy, which can be achieved by the degradation of cytoplasmic substituents. In addition, autophagy is crucial in the process of eliminating toxic proteins and defective organelles during the aging process [3]. There is increasing evidence that autophagy can influence cellular specialization, differentiation, protein trafficking, and unconventional secretion [4–6]. Autophagy is classified into three categories: Macroautophagy, microautophagy, and chaperone-mediated autophagy [7]. In this review, we focus on macroautophagy and refer to it simply as autophagy. The basic level of autophagy is required under normal conditions, but its induction can be mediated by metabolic changes [8, 9], oxidative stress [10], endoplasmic reticulum stress [11], mechanical damage [12, 13], and misfolded protein accumulation [14, 15]. Thus, it can be concluded that autophagy plays an important role in maintaining homeostasis in cells. Therefore, autophagy helps to recover amino acids and macromolecules for the synthesis of proteins and ATP, and improves cell stability and homeostasis by degrading cellular waste [16]. Changes in the internal and external environment of cells may occur during disease initiation and development and trigger autophagy to adapt cells to the new conditions. The autophagy mechanism has been associated with the pathogenesis of cancer [17, 18], cardiovascular diseases [19, 20], neurodegenerative diseases [21, 22], metabolic diseases [23, 24] and immune diseases [25]. The function of autophagy in diseases varies depending on the concept. It can induce/inhibit apoptosis and regulates other cellular events [26–29].

**Fig. 1:**
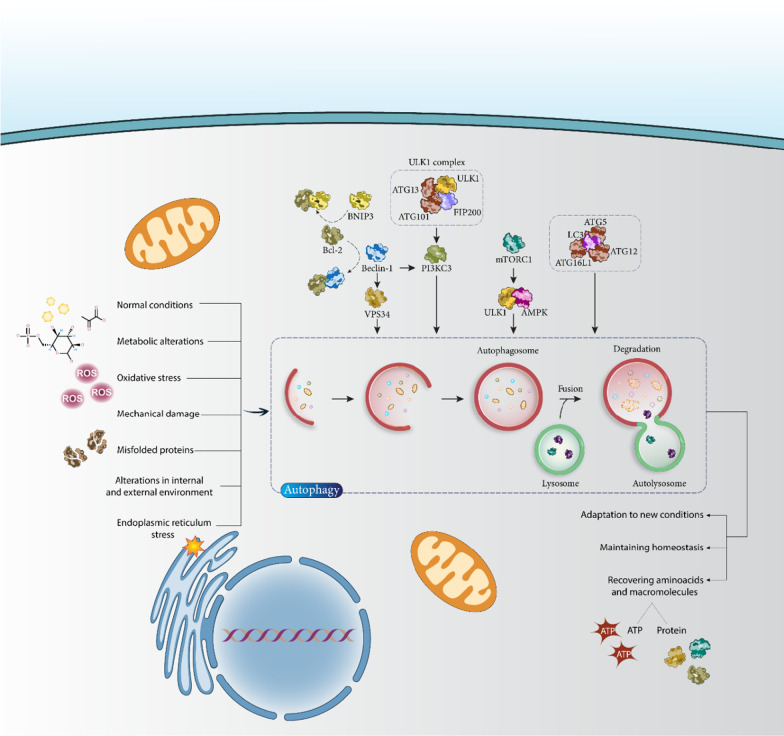
Regulation of autophagy in cells. ULK1, mTOR, ATGs, and Beclin-1 are the best known regulators of the autophagy mechanism in cells and therefore their targeting is of importance in the regulation of this molecular mechanism. In addition, oxidative stress, metabolic changes, and misfolded proteins are among the factors that can regulate the autophagy mechanism

Because autophagy is an important mechanism in cells, considerable efforts have been made to understand the molecular pathways that may regulate it. Figure 1 shows a schematic representation of the autophagy mechanism in cells. Autophagy is defined by the formation of double-membrane autophagosomes, which may be mediated by the function of the ULK1 complex containing ULK1, ATG13, FIP200, and ATG101 [30]. The ULK1 complex is able to induce PI3KC3 to stimulate autophagosome formation, and it also contributes to autophagosome nucleation and recruitment of autophagy-related proteins to the autophagosome membrane [31, 32]. ATG12-ATG5-ATG16L1 and LC3 are involved in the process of autophagosome elongation and maturation [33]. The interaction between AMPK and ULK1 is crucial for the induction of autophagy, which can be suppressed by mTORC1 when sufficient energy is available in cells [34–36]. During starvation, inhibition of mTORC1 occurs and AMPK signaling induces ULK1 in stimulating autophagy [37]. Beclin-1 is also a trigger of autophagy that interacts with the PI3KC3 complex. Moreover, Beclin-1 forms a complex with Bcl-2 as an anti-apoptotic protein, and when Bcl-2 binds to Beclin-1, it decreases the affinity of Beclin-1 for VPS34 to inhibit autophagy [38]. BNIP3 is a member of the Bcl-2 family that has a BH3 domain and releases Beclin-1 by disrupting the Bcl-2-Beclin-1 domain to initiate autophagy [39].

The role of autophagy in cancer is of great importance [40–42]. Upon stress, PKCeta increases autophagy and mediates senescence in breast tumor cells [43]. In a p53-dependent pathway, luteolin stimulates both apoptosis and autophagy [44]. It has been reported that inhibition of autophagy can improve the potential of photodynamic therapy in cancer [45]. In particular, gastric cancer stem cells stimulate autophagy to increase their survival rate, and thus can be considered as a potential therapeutic target [46]. Cx32 can increase the expression of AMPK to stimulate survival-promoting autophagy and thus mediate the resistance of cervical cancer cells to apoptosis [47]. During gastric cancer development, the lncRNA CCAT1 sponges miR-140-3p to increase ATG5 expression, stimulate autophagy, and promote tumor progression [48, 49]. Overexpression of PITX2 increases lung cancer progression in vitro and in vivo, and silencing of PITX2 increases autophagy, demonstrating that PITX2 suppresses autophagy in exerting oncogenic function in lung cancer [50]. DHOK, a new diterpene from the root of *Eurycoma longifolia Jack*, suppresses autophagy to exert anticancer activity in colorectal tumors [51]. On the other hand, autophagy can mediate the overexpression of miR-449a to prevent the proliferation and invasion of colorectal tumors [52, 53].

## Hepatocellular carcinoma: epidemiology, risk factors and oncogenic mechanisms

Primary liver cancer ranks seventh and second worldwide in incidence rate and mortality, respectively [54, 55]. Asia and Africa have the highest incidence rate of liver cancer [56]. The highest incidence rate of primary liver cancer is found in Mongolia (93.7 per 100,000) and the largest number of patients is found in China due to its high incidence rate (18.3 per 100,00) and the world's largest population (1.4 billion people) [55]. The predominant form of liver cancer is hepatocellular carcinoma (HCC), which accounts for up to 75% of all cases [56]. In many places where the incidence rate of HCC was high, it has been declining, and in areas with low incidence rates, an opposite trend has been observed [57]. Between 1978 and 2012, the incidence rate of HCC decreased in some areas such as Italy and Asian countries, while it increased in the United States, India, Oceania, and many European countries [57]. However, in recent years, a decrease in HCC cases has been observed in the United States [58, 59]. Factors that influence the likelihood of developing HCC include age, gender, and race. HCC shows a positive association with age up to 75 years [56]. The incidence rate of HCC is two to four times higher in men compared to women [56]. HCC is considered the sixth most common cancer worldwide and up to 500,000 new cases are diagnosed annually, which are responsible for a high mortality rate, making it the third leading cause of death among tumors [60, 61]. The incidence rate of HCC is estimated to be 3.6–10.5 per 100,000, which may increase to 16 per 100,000 worldwide [62, 63]. The survival rate of HCC patients is low, with 5% of them surviving more than 5 years after diagnosis. This is related to the late diagnosis of HCC patients and only 15% of patients are eligible for liver transplantation and surgery. 50% of them undergo non-surgical therapies and 35% or more receive the best treatment during diagnosis [63]. Risk factors for HCC vary and may include alcohol consumption, hepatitis virus infection, cirrhosis, and nonalcoholic fatty liver disease [64].

**Fig. 2:**
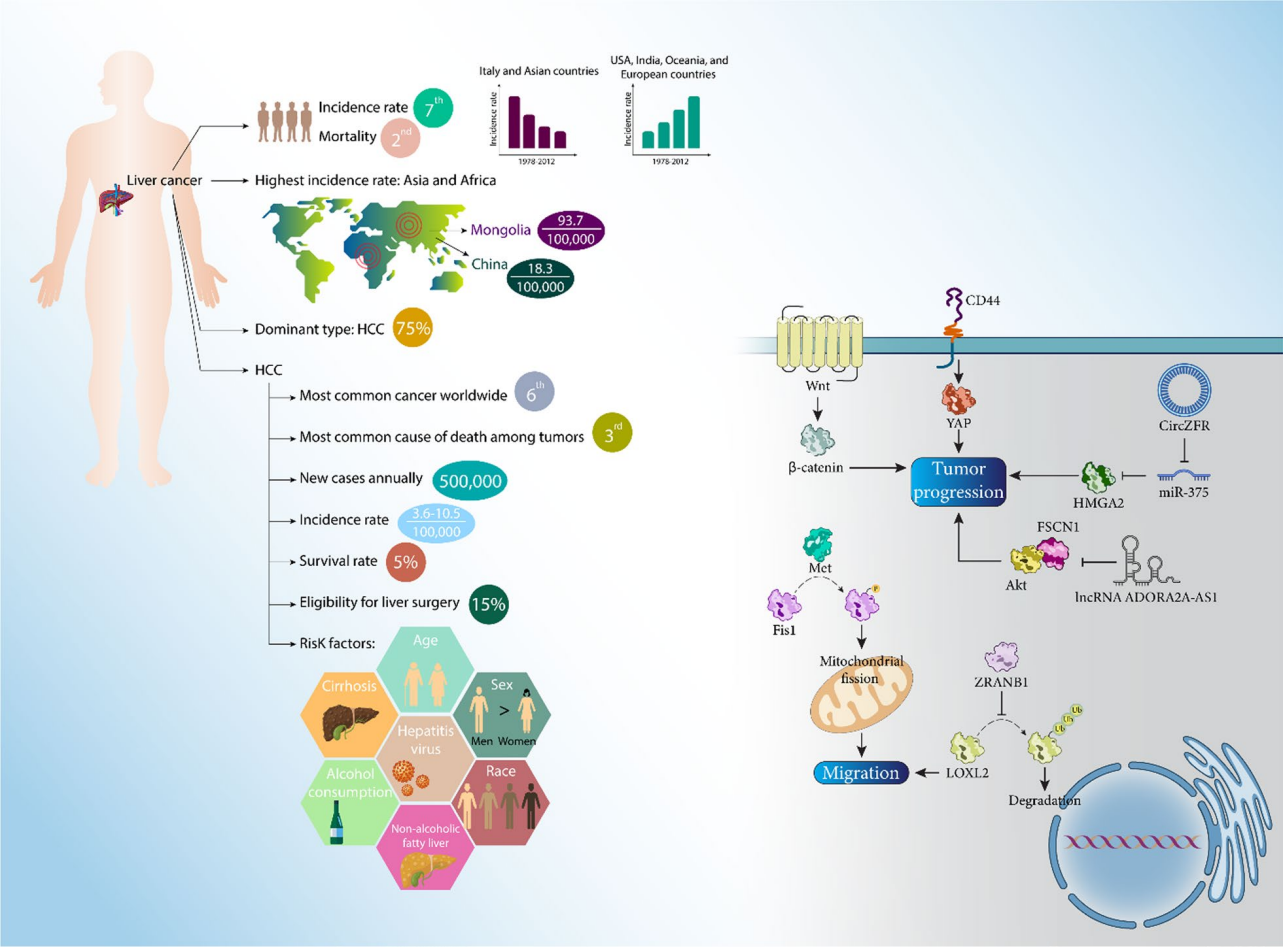
An overview of the risk factors for HCC and the pathways associated with progression

The progression of HCC depends on the interaction of some mechanisms and pathways at the molecular level. Phosphorylation of Fis1 occurs through Met to mediate mitochondrial fission and promote HCC migration [65]. CD44 can increase the expression of YAP to promote HCC progression [66]. It seems that obesity is a driving force for HCC progression. PI3Kγ ablation decreases HCC proliferation and reduces insulinemia, steatosis, and inflammatory cytokine concentration [67]. ZRANB1, as a deubiquitinate, is involved in the increasing progression and malignancy of HCC and, to this end, increases LOXL2 expression to promote tumor metastasis [68]. Stimulation of Wnt/β-catenin signaling has been shown to be associated with HCC progression [64], and DEAH-box polypeptide 32 induces β-catenin signaling to promote tumor growth [69]. CircZFR increases the expression of HMGA2 via miR-375 sponging, promoting HCC progression [70]. NTF3 has a low expression level in HCC and can be considered as a prognostic factor. Moreover, NFT3 increases the infiltration of immune cells including CD4+ cells, mast cells, macrophages, and natural killer cells in HCC [71]. On the other hand, there are factors that may affect the progression of HCC, such as ADORA2A-AS1, which suppresses the FSCN1/Akt axis to induce apoptosis and suppress tumor progression in vitro and in vivo [72]. Figure 2 shows a schematic representation of HCC progression and associated risk factors. Table 1 also provides an overview of the autophagy mechanism in HCC.

**Table 1:** An overview of the autophagy mechanism in HCC progression

Molecular pathway	Remark	Refs
TNFAIP8/Akt/mTOR	TNFAIP8 induces autophagy via inhibition of the Akt/mTOR axis	[220]
PI3K/Akt/mTOR	Suppression of the PI3K/Akt/mTOR axis by apigenin can induce autophagy and apoptosis Inhibition of autophagy enhances apoptosis	[221]
NBR2/JNK/Beclin1	LncRNA NBR2 reduces Beclin-1 expression via suppression of JNK and ERK1/2 signaling pathways in autophagy inhibition and impairs carcinogenesis	[222]
PNO1/MAPK	PNO1 induces autophagy and inhibits apoptosis via modulating MAPK signaling in HCC	[223]
BANCR/miR-590-5p/OLR1	Rutin promotes miR-590-5p expression via downregulation of BANCR to suppress OLR1 expression Inhibition of autophagy reverses drug resistance	[224]
PRDX1/Akt/mTOR	Natamycin increases the degradation of PRDX1 to inhibit the Akt/mTOR axis, leading to the induction of pro-survival autophagy	[225]
–	Inhibition of autophagy by nitric oxide increases apoptosis in tumor cells	[226]
JNK/Beclin-1	Tangerein increases phosphorylation of JNK to overexpress Beclin-1 to suppress tumor cell growth and metastasis	[227]
–	Inhibition of autophagy by daurisoline is critical for increasing cell death of tumor cells by cisplatin	[228]
ER stress/autophagy	ZNF263 promotes ER stress-induced autophagy in mediating apoptosis resistance	[229]
PRMT6/BAG/HSC70	PRMT6 reduces BAG5 expression to upregulate HSC70 in autophagy induction and increase tumor progression and survival	[230]
MEK/ERK/autophagy	Suppression of MEK/ERK axis leads to inhibition of autophagy and increase cytotoxicity of pemetrexed against tumor cells	[231]

## Targeting autophagy for regulating survival and proliferation rates

### Autophagy and metabolic reprogramming

HCC progression and development mainly depend on cellular energy metabolism, which is unique in tumor cells and shifts from oxidative phosphorylation to aerobic glycolysis, known as the Warburg effect [73]. Therefore, glycolysis not only contributes to the adequate energy supply of cancer cells, but also enables the immediate growth of cancer cells. The metabolic rate of glucose in HCC tissues is two times higher than that in paracarcinomatous tissues, and the ability of glycolysis in HCC was four times higher than oxidative phosphorylation [74]. The regulation of glycolysis in HCC is complicated and circRPN2 is an inhibitor of glycolysis and metastasis in HCC [75]. ZC3H13 inhibits glycolysis to promote drug sensitivity in HCC [76], and upregulation of HIF-1α by TFB2M can lead to induction of glycolysis [77]. Induction of autophagy in HCC may stimulate glycolysis to promote tumor progression. Autophagy induces nuclear translocation of β-catenin to increase the expression of MCT1, leading to induction of glycolysis in HCC [78].

### Autophagy and cell survival

Regulation of autophagy in HCC is closely related to tumor cell proliferation and survival. 3-hydroxybutyrate dehydrogenase 2 (BDH2) is downregulated in HCC and is responsible for reducing tumor cell progression. Poor expression of BDH2 in HCC mediates unfavorable prognosis, poor tumor differentiation, high tumor volume, and venous invasion. Overexpression of BDH2 stimulates apoptosis via the mitochondrial pathway and suppresses autophagy in impairing progression of HCC cells, indicating a pro-survival function of this factor [79]. One of the limitations of the studies is that they only focus on the role of molecular pathways in regulating autophagy and do not determine the exact function of autophagy in HCC. For example, exposure of HCC cells to paclitaxel results in a time- and concentration-dependent inhibition of proliferation and may enhance the formation of ROS and induction of autophagy. However, inhibition of autophagy by 3-MA promotes apoptosis in HCC cells [80].

The programmed death-ligand 1 (PD-L1)/PD-1 axis is considered an important target in cancer therapy because it can cause immune evasion and its suppression can promote the efficacy of immunotherapy. PD-L1 stimulates immune evasion to increase tumor proliferation and mediates poor prognosis [81, 82]. PD-1 is able to regulate the mTOR signaling pathway, which controls tumor growth [83–86]. PD-1 and PD-L1 are overexpressed in HCC and increase ATG13 expression to induce autophagy and increase the proliferation rate of tumor cells. It seems that the induction of autophagy in HCC may promote colony formation and cancer cell growth rate due to its pro-survival function. LncRNA ATB promotes the progression of HCC via the induction of autophagy. LncRNA ATB promotes ATG54 expression via Yes-associated protein (YAP) induction to mediate autophagy [87].

**Fig. 3:**
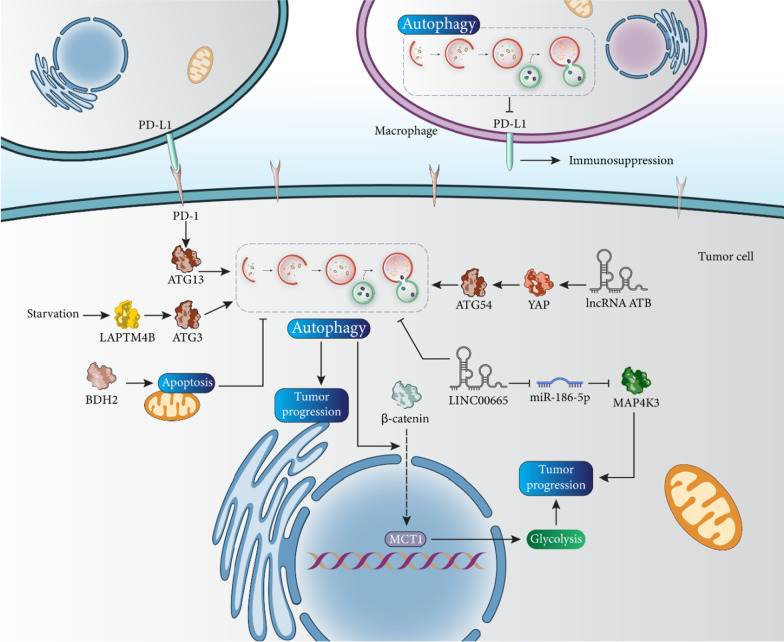
Targeting autophagy to regulate the survival rate of HCC cells. PD-L1 is involved in the process of immunosuppression, while autophagy inhibits PD-L1 in enhancing cancer immunity in HCC. More importantly, pro-survival autophagy can lead to the activation of Wnt signaling by mediating the nuclear translocation of β-catenin, which induces glycolysis and promotes HCC progression

Impairment of autophagy in macrophages may promote the process of tumorigenesis in HCC, which has been confirmed in vivo in mice. Loss of autophagy in macrophages leads to overexpression of PD -L1 to mediate immunosuppression in HCC and promotes HCC progression and carcinogenesis [88]. During starvation, the proliferation rate of HCC cells is increased by LAPTM4B. Specifically, LAPTM4B promotes ATG3 expression to induce autophagy, which is important for inhibiting apoptosis and improving HCC cell viability [89]. An interesting study has investigated the interaction and crosstalk of apoptosis and autophagy in HCC and demonstrated that inhibition of autophagy can induce apoptosis and reduce the proliferation rate of tumor cells exposed to photothermal therapy [90]. Therefore, targeting autophagy is important for regulating proliferation and tumorigenesis in HCC, and the function of autophagy can be tumor-promoting or tumor-suppressing (Fig. 3) [91–96].

### Autophagy and apoptosis interaction

Another limitation of the studies is that some of them did not investigate the interaction between autophagy and apoptosis in HCC. According to studies, autophagy and apoptosis show interactions in normal and cancerous cells and even in different diseases, and autophagy can sometimes inhibit apoptosis [97–99]. However, this role has been ignored in some studies on HCC. LINC00665 reduces the expression of miR-186-5p to upregulate MAP4K3 to increase the survival rate of HCC. Silencing LINC00665 decreases cancer cell viability and stimulates apoptosis and autophagy [100].

The current section provides new insights into the role of autophagy in HCC tumorigenesis, focusing on the molecular signaling pathways involved. Surprisingly, one of the major pathways regulating autophagy is PD-L1, a regulator of immune escape in tumor cells, which can induce autophagy in HCC tumorigenesis. The interaction of autophagy in enhancing HCC progression can be summarized in several aspects. The first is the inhibition of apoptosis by survival-promoting autophagy, which supports HCC cell progression. Stimulation of autophagy in HCC favors colony formation and promotes tumor cell proliferation. Another aspect is that metabolic reprogramming in HCC can be regulated by autophagy and this mechanism can promote glycolysis to accelerate the progression of HCC.

## Targeting autophagy for regulating metastasis

### General description of metastasis in HCC

Metastasis has been a problem in clinical management of cancer. More than 90% of tumor-related deaths are due to metastasis [101]. The reason that metastasis leads to high mortality in cancer patients is the inefficiency of surgery in treating metastatic tumor and the low effectiveness of chemotherapy and radiotherapy at this stage. Metastasis is a complicated process in which tumor cells migrate from their primary site and spread to other parts of the body to survive, adapt to conditions in a new tumor microenvironment, proliferate, and form new colonies at distant sites. The cellular and molecular interactions that occur in the tumor microenvironment greatly influence the migration and invasion potential of cancer cells [102–104]. In HCC, the process of metastasis is similarly complex as in other tumor types, and this complexity may complicate the prevention of tumor cell invasion. Secretion of the cytokine IL-9 may lead to increased invasion of HCC due to stimulation of the JAK2/signal transducer and activator of transcription 3 (STAT3) axis [105]. AP-2β reduces the expression of Snail and Slug to suppress epithelial-mesenchymal transition (EMT) and decrease the metastasis of HCC [106]. In addition, the factors associated with metastasis in HCC may mediate tumor recurrence, indicating that the prevention and management of metastasis are important for improving the survival rate of cancer patients [107].

### An overview of autophagy and metastasis in HCC

The rapid growth of tumor cells may lead to insufficient blood supply and hypoxia in the microenvironment of solid tumors [108, 109]. Then, cancer cells are forced to adapt to the new conditions by metastasis. During hypoxia, autophagy is induced to remove damaged organelles, modulate the formation of reactive oxygen species (ROS) and reduce apoptosis [110]. Even in the absence of apoptosis, the presence of autophagy is critical for cancer cell survival during hypoxia [111–113]. Overexpression of YTHDF1 by hypoxia inducible factor-1α (HIF-1α) during hypoxia may increase HCC cell progression, including proliferation and metastasis, and stimulate autophagy. Overexpressed YTHDF1 increases the expression of ATG2A and ATG14 to induce autophagy and promote metastasis of HCC cells [114]. However, induction of autophagy does not always promote increasing metastasis in HCC. SOCS protein is a modulator of cytokine signaling [115–117] and dysregulated expression of SOCS has been associated with disease pathogenesis and progression [118]. Low expression of SOCS5 may impair metastasis of HCC cells. The expression of SOCS5 in HCC cells and tissues is thought to be high and worsen the prognosis and survival of patients. Up-regulation of SOCS5 leads to induction of the PI3K/Akt/mTOR axis to reduce the expression of Beclin-1 in autophagy inhibition. However, downregulation of SOCS5 induces autophagy via suppression of the PI3K/Akt/mTOR axis to impede HCC cell invasion [91]. Therefore, the interplay between autophagy and metastasis is critical for HCC progression [119].

CCAT2 is located on chromosome 8q24.21 and is considered an oncogenic lncRNA in cancer [120]. CCAT2 mediates unfavorable prognosis in HCC [121] and may increase the expression of NDRG1 and MDM2 in promoting tumor progression [122, 123]. Overexpression of CCAT2 is observed in HCC samples and leads to advanced stage and venous invasion. CCAT2 decreases the expression of miR-4496 in the cytoplasm to promote ATG expression, trigger autophagy, and enhance metastasis of HCC cells [124]. One of the best-known regulators of autophagy is Beclin-1, which was mentioned in the introduction. BMP4 stimulates c-Jun N-terminal kinase (JNK) signaling to increase the expression of Beclin-1 in autophagy induction, leading to a marked increase in HCC cell metastasis and invasion [125].

Glycochenodeoxycholate (GCDC) is one of the components of bile acid and can cause liver damage due to its hydrophobic acidic nature [80, 126–130]. Hydrophobic bile acid can lead to apoptosis and necrosis in hepatocytes [131]. In HCC, GCDC has been associated with an increase in progression and metastasis of HCC cells. GCDC promotes AMPK expression to inhibit mTOR signaling, leading to induction of autophagy and increased tumor cell invasion [132]. It appears that a lack of autophagy, which may be mediated by caveolin-1, leads to induction of angiogenesis and acceleration of metastasis in HCC [133].

### Autophagy and EMT

EMT is the closest mechanism associated with tumor cell invasion and metastasis, as well as their resistance to therapy, and is characterized by morphological and physiological changes such as loss of cell polarity, disruption of intracellular junctions, increase in growth and invasion, increase in N-cadherin and vimentin levels, and decrease in E-cadherin [134–137]. Fluid shear stress (FSS) increases autophagosome formation and promotes the expression of Beclin-1, ATG7, and LC3II to mediate EMT and enhance HCC cell metastasis [138].

### Autophagy and anoikis

**Fig. 4:**
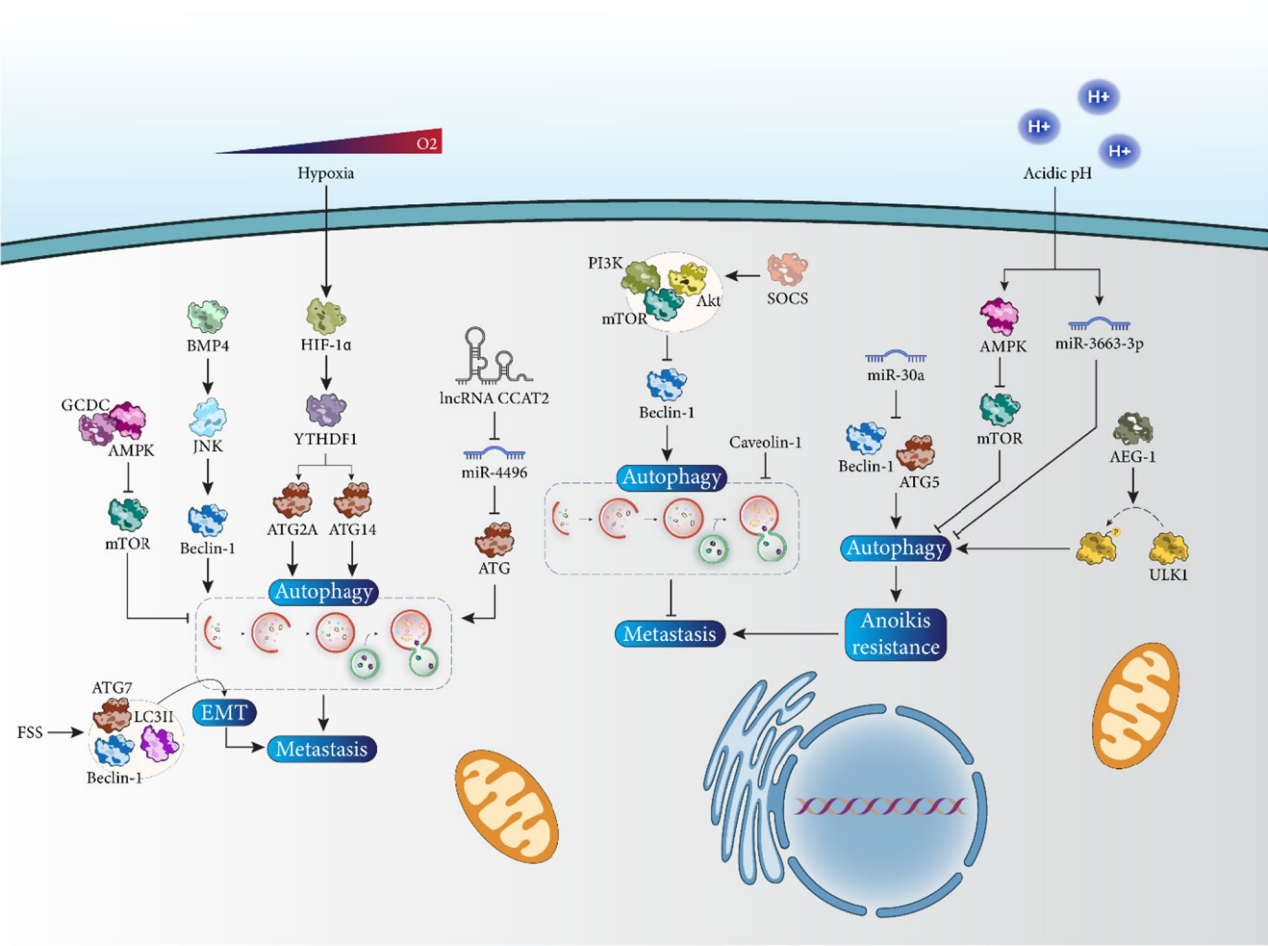
Targeting autophagy to regulate metastasis in HCC. Anoikis resistance and EMT as the two most important factors in regulating HCC cell invasion are influenced by autophagy mechanism. When autophagy has an oncogenic function, its induction by Beclin-1 and AMPK/mTOR signaling pathway may lead to EMT induction, which promotes tumor metastasis

Anoikis is a type of detachment from the extracellular matrix (ECM) [139] and the development of resistance to anoikis is critical for cancer cells to enhance their invasion and metastasis [140]. During anoikis resistance, there may be activation of autophagy, which is responsible for adaptation to stressful conditions such as oxidative stress, starvation, hypoxia, and metabolic reprogramming [141]. There is growing evidence of a link between autophagy and anoikis resistance in HCC. Acidic extracellular pH inhibits mTOR signaling via upregulation of AMP-protein kinase (AMPK) expression to induce autophagy. Moreover, miR-3663-3p is downregulated at acidic extracellular pH to induce autophagy, which promotes anoikis resistance in HCC [142]. Therefore, autophagy stimulates anoikis resistance in increasing progression and metastasis of HCC cells. miR-30a reduces the levels of Beclin-1 and ATG5 to suppress autophagy and anoikis resistance and limit metastasis of HCC cells [143]. AEG-1 stimulates phosphorylation of ULK1 to stimulate autophagy in mediating anoikis resistance and enhancing HCC cell invasion [144]. Based on these studies, targeting autophagy and related molecular signaling pathways is of importance in treating HCC and reducing cancer cell metastasis (Fig. 4) [145].

Regulation of proliferation by autophagy has shown that interfering with this mechanism may provide new insights into how tumor survival is affected. Most importantly, autophagy has been found to be related to metastasis as another feature of HCC. Autophagy may act as a trigger/inhibitor of metastasis in HCC. Overexpression of ATG2A and ATG14 leads to autophagy and a subsequent increase in metastasis of HCC cells, whereas inhibition of the Beclin-1/autophagy axis by SOCS5 promotes metastasis. Since metastasis is closely related to the malignancy of tumor cells and may also mediate chemoresistance, induction of autophagy in HCC cells, when acting as a tumor suppressor, may contribute significantly to the impairment of carcinogenesis.

## Targeting autophagy for regulating drug resistance

### Basic evolution of drug resistance in HCC

The process of chemoresistance in HCC has been challenging in recent years, and prevention of mitochondrial respiration and stimulation of oxidative stress have been responsible for overcoming chemoresistance [146]. Overexpression of ICMT prevents apoptosis and induces doxorubicin resistance in HCC [147]. Overexpression of Nrf2 induces chemoresistance in HCC and metformin suppresses Nrf2-mediated glycolysis, thereby increasing the drug sensitivity of cancer cells [148]. Downregulation of USP7 reduces HCC cell growth and metastasis and is responsible for drug sensitivity [149]. Glyochenodeoxycholic acid stimulates STAT3 signaling to promote stem cell formation of HCC cells and mediates drug resistance [150]. Therefore, molecular pathways contribute to HCC cell progression and mediate drug resistance, so the role of autophagy mechanism in this aspect is under investigation.

### Related molecular pathways

Upregulation of CD24 is observed in HCC cells and those resistant to sorafenib chemotherapy. CD24 increases the expression of PP2A and suppresses the mTOR/Akt axis to induce autophagy, which triggers sorafenib resistance in HCC [151]. The redox status of tumor cells is different from that of normal cells [152], and overexpression of antioxidant factors can enhance the progression of HCC cells [153]. Ferroptosis is a type of programmed oxidative cell death characterized by stimulation of the Fenton reaction, which promotes the formation of ROS and increases the accumulation of lipid peroxidation products [154]. Sorafenib can cause depletion of GSH, triggering ferroptosis in HCC [155, 156]. Overexpression of CISD2 in HCC is responsible for sorafenib resistance in tumor cells. Silencing of CISD2 stimulates apoptosis and uncontrolled autophagy in HCC and increases sorafenib-mediated ferroptosis in tumor cells [157]. Inhibition of pro-survival autophagy is important for increasing sorafenib sensitivity of HCC cells. Downregulation of miR-541 in HCC leads to sorafenib resistance and suppresses proliferation, metastasis, and autophagy in vitro and in vivo. miR-541 decreases ATG2A and RAB1B levels to inhibit autophagy and increase sorafenib sensitivity of HCC cells [158]. Although the function of miR-541 is critical for enhancing drug sensitivity in HCC, upregulation of miR-25 leads to sorafenib resistance. miR-25 decreases FBXW7 expression to trigger autophagy in mediating sorafenib resistance in HCC [159].

The expression of Rage is found in various types of cells [160] and has been found to be associated with the progression of inflammatory diseases such as cancer [160–162]. Recently, the role of Rage in regulating the progression of HCC cells has been investigated. CircRNA-101368 increases Rage levels and thus promotes HCC progression [163]. The upregulation of Rage in HCC promotes the growth rate of HCC cells and stimulates sorafenib resistance. Loss of Rage expression leads to stimulation of AMPK signaling to reduce mTOR expression in autophagy induction and promote sorafenib response in HCC [164]. Yes-associated protein (YAP) is one of the new targets in HCC therapy and KAT6A increases the expression of YAP, which promotes HCC progression and mediates sorafenib resistance [165]. Cinacalcet inhibits the YAP /TAZ axis to prevent HCC progression [166]. Moreover, overexpression of YAP leads to EMT induction, which enhances HCC metastasis [167]. An experiment has shown that overexpression of YAP can lead to drug resistance in HCC. Inhibition of YAP increases drug sensitivity in HCC via mediating autophagy-induced cell death. Knockdown of YAP leads to increased RAC1-mediated ROS generation to suppress mTOR signaling, resulting in autophagy induction and chemosensitivity in HCC [168].

### Autophagy and apoptosis in chemoresistance

The major pathway by which chemotherapeutic agents exert cytotoxicity on tumor cells is the induction of apoptosis, which can be stimulated by both mitochondria and endoplasmic reticulum stress (ER). ER Stress leads to activation of the UPR, which can mediate apoptosis [169, 170]. Moreover, ER stress can induce both apoptosis and autophagy, and compounds targeting ER stress are of interest for the treatment of disease and cancer [171]. In HCC cells exposed to sorafenib, overexpression of IRE1 is critical for the induction of autophagy via the ER stress signaling pathway. Moreover, activation of autophagy reduces ER stress-induced apoptosis in HCC. Therefore, inhibition of autophagy and its targeting may improve the potential of chemotherapeutic agents in apoptosis induction in HCC [172–174]. Table 2 summarizes the role of autophagy in drug resistance in HCC.

**Table 2:** Targeting autophagy for drug sensitivity in HCC

Molecular pathway	Drug	Remark	Refs
m6A modification/FOXO3	Sorafenib	Enhanced stability of FOXO3 by m6A modification in inducing autophagy and mediating chemoresistance	[232]
HGF-MET	–	HFG- MET induces autophagy in mediating drug resistance Inhibition of HGF-MET and autophagy can reduce tumor progression and mediates drug sensitivity	[233]
Hypoxia/FOXO3a	Sorafenib	Hypoxia is able to increase the expression of FOXO3a in autophagy induction and mediate drug resistance	[234]
HMGB1/AMPK/mTOR	Doxorubicin	HMGB1 increases AMPK expression and inhibits mTOR Autophagy induction Doxorubicin resistance	[235]
Osteopontin/FoxO3/autophagy	Epirubicin Cisplatin	Osteopontin increases FoxO3 stability in autophagy induction and mediation of drug resistance	[236]
–	Oxaliplatin	Tumor-associated macrophages induce autophagy and inhibit apoptosis in mediating drug resistance	[237]
miR-26b/USP9X/p53/autophagy	Doxorubicin	miR-26b reduces USP9X expression to prevent p53 degradation in autophagy inhibition and desensitization to doxorubicin chemotherapy	[238]
SNHG16/miR-23b-3p/EGF1	Sorafenib	SNHG16 increases EGF1 expression via inhibition of miR-23b-3p in autophagy induction and apoptosis inhibition, leading to the development of drug resistance	[239]
Egr-1	–	Upregulation of Egr-1 during hypoxia and its positive association with autophagy in triggering chemoresistance	[240]
miR-21/PTEN/Akt	Sorafenib	miR-21 increases Akt expression by inhibiting PTEN signaling Inhibition of autophagy Development of drug resistance	[241]
cFLIP/ER stress	Sorafenib	Silencing of cFLIP impairs drug resistance by preventing ER stress-mediated autophagy	[242]
Beclin-1	KU-0063794	Silencing of Beclin-1 suppresses autophagy and increases drug sensitivity	[243]
CD13/p38/Hsp27/CREB/ATG7	5-flourouracil	CD13 increases ATG7 expression via the p38/Hsp27/CREB axis, which induces chemoresistance	[244]
miR-23b-3p/ATG12	Sorafenib	miR-23b-3p reduces ATG12 expression, impairing autophagy and reversing sorafenib resistance	[245]
p57/autophagy	EGFR inhibitors	p57 induces autophagy and enhances the potential of EGFR inhibitors in combating HCC	[246]
VRK2	JNK1/MAPK8/Bcl-2/Beclin-1	VRK2 induces Beclin-1 expression via activation of the JNK1/MAPK8 axis to mediate dissociation of Bcl-2 from Beclin-1, triggering autophagy and mediating drug resistance	[247]
Akt	Sorafenib	Suppression of Akt signaling reverses autophagy that promotes survival to autophagy that promotes death, thereby reversing drug resistance	[248]
miR-101-3p/Beclin-1	oxaliplatin	miR-101-3p reduces the expression of Beclin-1 in inhibiting autophagy and reversing drug resistance	[249]
miR-651-3p/ATG3	Cisplatin	miR-651-3p reduces ATG3 expression in suppressing autophagy and chemoresistance	[250]

The process of chemoresistance is highly complicated and it is impossible to target all the molecular pathways involved in this condition, but it is possible to adopt some of the most important mechanisms in this case. Because of the interaction of autophagy with other cell death mechanisms, its induction may mediate ferroptosis in enhancing drug sensitivity, while inhibition of autophagy by miR-541 is critical in preventing chemoresistance, again demonstrating the dual function of autophagy in this case. One of the drawbacks of the current studies is that they did not focus on other chemotherapeutic agents such as doxorubicin, paclitaxel, and others, but focused on sorafenib. Future studies should therefore pay particular attention to the role of autophagy in the resistance of HCC to the above agents.

## Targeting autophagy for regulating radioresistance

**Fig. 5:**
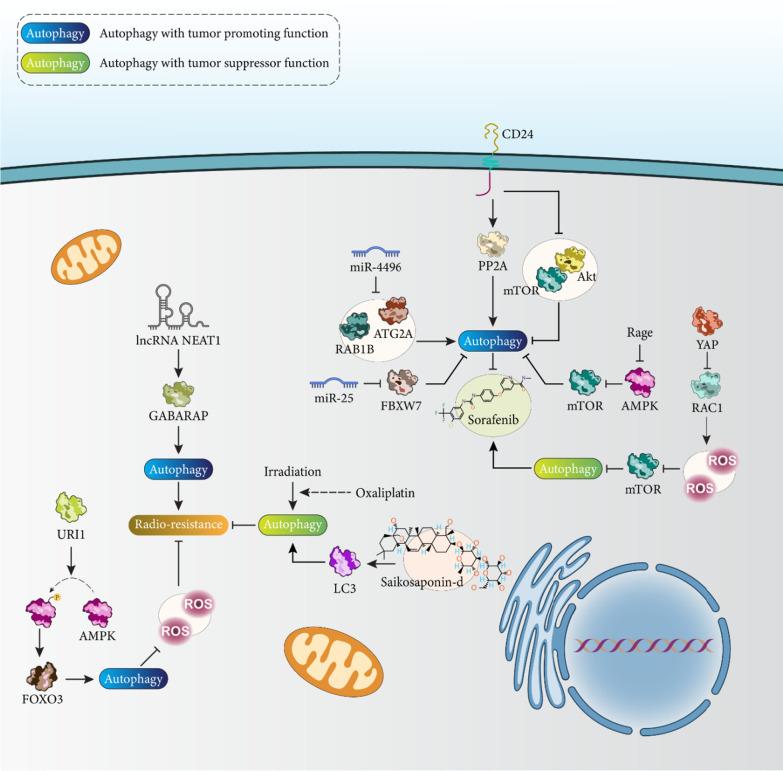
Autophagy, drug resistance, and radioresistance in HCC. Based on the function of autophagy in regulating proliferation and metastasis of HCC cells, this metabolic pathway may be involved in regulating radio- and chemoresistance of HCC cells. The resistance of HCC cells to sorafenib and oxaliplatin is tightly regulated by the autophagy mechanism. Moreover, activation of autophagy by NEAT1 may lead to radioresistance. More importantly, autophagy can reduce the levels of ROS in triggering radioresistance

Modulation of radioresistance in HCC is of importance because it is a conventional therapy for HCC. If the potential of therapy is reduced due to resistance, a functional analysis of genes should be performed to reveal the role of factors involved in progression and resistance. Radiotherapy stimulates CD8+ T cell function to impair HCC progression, and sorafenib is considered an inhibitor of radioresistance [175]. HCC cells overexpressing γ GCSh prevent apoptosis, which is beneficial for inducing radioresistance [176]. This section focuses on the role of autophagy in regulating radioresistance in HCC. NEAT1 is a regulator of autophagy in cancer and its downregulation can stimulate autophagy and ferroptosis [177]. The upregulation of NEAT1 in HCC has been associated with an important property known as radioresistance. This effect is mediated by overexpression of gamma-aminobutyric acid receptor-associated protein (GABARAP) and induction of autophagy [178]. The effect of autophagy on radioresistance in HCC is uncertain. Irradiation of HCC cells leads to autophagy induction, and oxaliplatin promotes autophagy activation by increasing the cytotoxicity of radiotherapy in the treatment of HCC [179]. However, most studies have focused on the role of autophagy as a pro-survival mechanism in triggering radioresistance. ASPP is an apoptosis regulatory protein and has three members, including ASPP1, ASPP2, and iASPP. ASPP2 is downregulated in HCC due to its methylation [180, 181]. Moreover, ASPP2 prevents autophagy to increase cell response to RAS [182]. Downregulation of ASPP2 may lead to an increase in HCC cell survival. Low expression of ASPP2 leads to overexpression of Beclin-1 and induction of autophagy [183]. Previous experiments have investigated the role of ASPP2 in the regulation of autophagy and its association with drug resistance, and future studies may be warranted to investigate its role in radioresistance. RAD001 is an inhibitor of mTOR signaling that stimulates autophagy as a pro-death mechanism to enhance autophagy and the efficacy of radiotherapy in combating HCC [184]. Saikosaponin-d promotes LC3 levels and increases autophagosome formation to stimulate autophagy and increase the radiosensitivity of HCC cells [185]. The biological effects of irradiation are mediated by the formation of ROS [186]. Irradiation can increase the formation of ROS and mediates oxidative stress [187]. Thus, when the levels of ROS decrease, the sensitivity of HCC cells to radiotherapy decreases. URI1 induces AMPK phosphorylation to increase forkhead box O3 (FOXO3) levels to trigger autophagy via increased autophagosome formation and prevent ROS formation by radiotherapy in HCC [188]. According to these studies, targeting autophagy and regulating related molecular signaling pathways are important for modulating the response of HCC cells to radiotherapy (Fig. 5) [189–193].

Although the focus of many studies has been on drug resistance, there have also been attempts to demonstrate the role of autophagy in mediating radioresistance in HCC. Most studies show that induction of cytotoxic autophagy is important in mediating radiosensitivity. One of the most important aspects is the use of chemotherapeutic agents such as oxaliplatin together with radiotherapy to stimulate pro-death autophagy and reduce HCC progression.

## Anticancer agents modulating autophagy in hepatocellular carcinoma

### Synthetic drugs

The use of anticancer agents, other than chemotherapeutic agents, is an increasing trend in the treatment of HCC as resistance to conventional therapies develops [194–196]. Shikonin is an inhibitor of HCC progression and suppresses the PI3K/Akt/mTOR axis to stimulate both apoptosis and autophagy to reduce cancer cell malignancy [197]. However, when an anti-cancer agent stimulates autophagy, it does not mean that autophagy promotes tumor progression. Thus, in HCC, myricetin stimulates both apoptosis and autophagy by affecting endoplasmic reticulum (ER) stress. Although activation of ER stress by myricetin stimulates apoptosis to reduce HCC progression, activation of autophagy is a supportive mechanism, and its inhibition may enhance the anticancer effect of myricetin anticancer therapy [198].

Imatinib is a tyrosine kinase inhibitor and impairs HCC cell metastasis by increasing NM23 expression (199). To improve the anti-cancer activity of imatinib against HCC, attempts have been made to combine it with other antitumor agents such as sulfasalazine and GNF-5 [200, 201]. Moreover, incorporation of imatinib into lactoferrin-modified PEGylated liquid crystalline nanostructures induces apoptosis in HCC via the mitochondrial pathway [202]. Imatinib prevents phosphorylation of protein kinase B (Akt) and promotes expression of p62 and suppresses progression of HCC in vitro and in vivo. Imatinib impairs HCC progression via regulating the above signaling pathways to inhibit autophagy [203].

### Natural compounds

Another antitumor agent that is popular in HCC treatment is dioscin. Dioscin suppresses TGF-β1-induced EMT in HCC to reduce cancer cell metastasis [204]. In addition, dioscin increases Bax and caspase-3 levels and decreases Bcl-2 levels in apoptosis induction in HCC [205]. Dioscin stimulates apoptosis, autophagy, and DNA damage in HCC cells and reduces growth and metastasis. Dioscin increases the levels of Beclin-1 and LC-3 and decreases the levels of p-Akt and p-mTOR in inducing autophagy and promoting progression of HCC cells [206]. Another strategy is the simultaneous use of two anticancer drugs. For example, in one experiment, costunolide (CL) and dehydrocostuslactone (DCL) were used as two bioactive components of an extract of sesquiterpene lactones to treat HCC. CL and DCL promote the accumulation of LC3 and p62 to suppress autophagy and prevent HCC progression [207]. In the previous sections, it has been shown that inhibition of autophagy may be beneficial in improving apoptosis induction in HCC. However, when autophagy has a death-promoting function, its inhibition decreases apoptosis. Isoqerucetin stimulates the AMPK/mTOR/p70S6K axis to mediate apoptosis and autophagy in HCC. Inhibition of autophagy decreases apoptosis by lowering the Bax/Bcl-2 ratio and preventing capase-3 activation and PARP cleavage, confirming the anticancer effect of autophagy in HCC [208].

The most controversial part is that the function of autophagy in HCC is completely different depending on the conditions. For example, a previous experiment showed that imatinib suppresses autophagy in HCC therapy [203]. However, another experiment shows that ursodeoxycholic acid promotes LC3B expression to stimulate autophagy and thus prevent HCC progression [209]. Moreover, both studies have shown that autophagy is regulated in vitro and in vivo and has an effect on the progression of HCC.

**Fig. 6:**
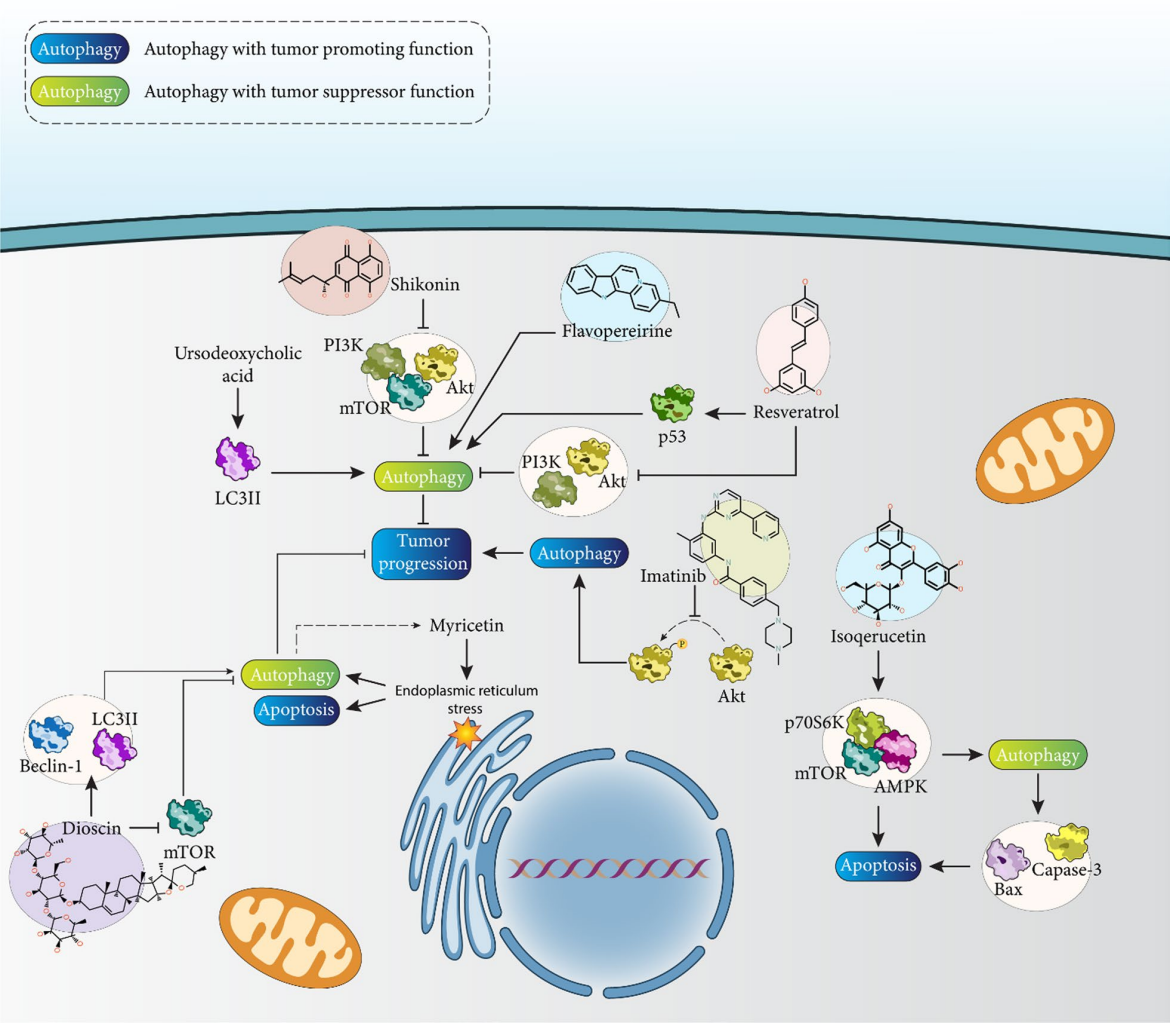
Regulation of autophagy by anti-cancer agents in the treatment of HCC. Molecular pathways related to autophagy, such as PI3K/Akt/mTOR, AMPK, and LC3II, are regulated by anticancer drugs. Of note, induction of autophagy by antitumor agents not only has a pro-death function, but sometimes also a pro-survival function, so in this case, inhibition of autophagy increases the potential of antitumor agents to induce apoptosis

Resveratrol is an effective anti-cancer agent against HCC and suppresses Akt signaling by increasing phosphatase and tensin homolog (PTEN) expression, thereby reducing the malignancy of HCC [210]. Resveratrol promotes anti-tumor immunity in HCC by reducing the number of CD8+ CD122+ Treg cells [211]. Moreover, resveratrol reduces Gli-1 expression in HCC suppression [212]. Resveratrol is able to upregulate the expression of p53 and suppress the PI3K/Akt signaling pathway to induce autophagy to prevent the progression of HCC [213]. Moreover, flavopereirine induces autophagy to prevent the progression of HCC [214]. Based on these studies, most of the anti-tumor agents targeting autophagy (induction or inhibition) in the treatment of HCC are natural products and have shown promising results [215–217]. However, natural products have poor bioavailability [218, 219], and their future application may be facilitated by using nanostructures for their delivery in the treatment of HCC and modulation of autophagy (Fig. 6; Table 3).

**Table 3:** Use of anticancer compounds for targeting autophagy in HCC treatment

Anticancer compound	Molecular pathway	Remark	Ref
Sterodial saponins	LC3-II p62	Sterodial saponins increase LC3- II and p62 levels, indicating their role in impairing autophagy flux and reducing tumor growth	[251]
Quercetin	Akt/mTOR	Quercetin inhibits Akt/mTOR axis to induce autophagy and mediate apoptosis	[252]
Propyl gallate	-	Increasing the conversion of LC3-I to LC3- II and triggering autophagy in reducing HCC progression	[253]
Lycorine	TCPR1/Akt/mTOR	Lycorine suppresses the TCPR1/Akt/mTOR axis in triggering autophagy and apoptosis	[254]
Exenatide	mTOR	Exenatide suppresses the mTOR pathway in triggering apoptosis and autophagy	[255]
Astragaloside II	MAPK/mTOR	Astragaloside II suppresses autophagy and increases sensitivity to 5-fluorouracil Induction of MAPK/mTOR signaling	[256]
Metformin	AMPK	Metformin increases sorafenib sensitivity of HCC cells via autophagy induction in an AMPK-dependent manner	[257]
Regorafenib	Akt/mTOR	Regorafenib suppresses the Akt/mTOR axis by inducing autophagy and reducing tumor cell growth rate	[258]
4-phenoxyphenol derivative, 4-[4-(4-hydroxyphenoxy)phenoxy]phenol (4-HPPP)	α-tubulin	4-HPPP reduces α-tubulin expression and induces apoptosis and autophagy	[259]
Wogonin Sorafenib	–	Combination of wogonin and sorafenib induces apoptosis and suppresses autophagy to reduce tumor progression	[260]
ABT-737	Beclin-1	ABT-737 induces autophagy via ROS and upregulation of Beclin-1 Pro-survival function of autophagy	[261]
3-methyladenine	–	Inhibition of autophagy in enhancing sorafenib sensitivity of cancer cells	[262]
Cardiac glycosides	AMPK	Autophagy induction by AMPK upregulation Autophagy inhibition increases capacity for apoptosis induction	[263]
Morin hydrate	PARP1/HMGB1	Morin hydrate suppresses PARP1/HMGB1 axis in autophagy inhibition and suppresses cisplatin resistance	[264]
Chloroquine	–	Chloroquine suppresses autophagy and prevents mitochondrial dysfunction in promoting chemosensitivity	[265]
GL-V9	JNK2	GL-V9 suppresses autophagy via downregulation of JNK2 in reversing adriamycin resistance	[266]

This section has shown that modulation of autophagy by synthetic and natural agents offers a great opportunity for improved efficacy in the treatment of HCC. Sometimes, autophagy induced by anti-tumor agents has a pro-survival function, and in this case, inhibition of autophagy is suggested to enhance its efficacy in cancer therapy. One of the gaps in the current field is that studies have ignored the role of small molecules in regulating autophagy in HCC. Since the molecular signaling pathways related to autophagy such as ATGs, Beclin-1, and AMPK have been recognized and their structure has also been understood, it is highly recommended to develop new small molecules to affect autophagy in HCC therapy in the near future.

## Conclusion and remarks

The autophagy mechanism is a molecular event in normal and cancer cells that has a completely different function depending on the context. In normal cells, the goal of autophagy is to break down aged organelles and decompose toxins. Therefore, a baseline level of autophagy or its induction may be beneficial for maintaining homeostasis under physiological conditions. However, as cancer cells progress, they may induce or inhibit autophagy depending on their condition to improve their survival rate. Since HCC is the most malignant and lethal liver cancer, the influence of autophagy on tumor cell progression needs to be emphasized. Autophagy has two distinct functions in HCC that impact survival and death. In the context of the pro-survival function, induction of autophagy can significantly enhance progression and viability of HCC cells, whereas pro-death autophagy impairs tumor progression. ER stress can mediate both apoptosis and autophagy in HCC cells, and inhibition of pro-survival autophagy has been shown to be beneficial in increasing ER stress-mediated apoptosis in tumor cells. AMPK, Beclin-1, and the Akt/mTOR pathway are the major regulators of autophagy that have been studied in HCC. Autophagy can increase HCC cell viability and prevent apoptosis, whereas death-promoting autophagy exerts a different function. Activation of autophagy may lead to increased invasion of HCC cells and induce EMT, whereas anti-cancer autophagy suppresses tumor cell migration. In addition, targeting autophagy is of interest to increase the sensitivity of HCC cells to drugs and radioactivity. Quercetin, resveratrol, and lycorine are among the compounds that target autophagy in the treatment of HCC. Induction of autophagy by anticancer agents does not indicate their tumor-suppressive effects, and sometimes inhibition of autophagy may improve the cytotoxicity of these agents in combating HCC. Future application of these findings in the clinic could greatly improve the prognosis, survival, and ability to treat HCC patients.

Basic research is important when it is translated into clinical practice in the treatment of patients. The current situation in the treatment of HCC patients is complicated because physicians face various problems in treating patients. Regardless of the lack of specific and highly sensitive tools for early diagnosis of HCC patients, their diagnosis in advanced and metastatic stages leads to difficulties in treatment, especially due to resistance to therapy. The clinical application of autophagy in these patients is that autophagy can be assessed prior to therapy to predict tumor cell response, and then a more effective therapeutic regimen can be applied to patients. Furthermore, since gene therapy has only recently entered the field of cancer therapy, it is possible that in the near future, factors related to autophagy can be targeted and modulated to enable more effective treatment of cancer patients.

## Data Availability

Not applicable.
